# Post-transcriptional control of gene expression following stress: the role of RNA-binding proteins

**DOI:** 10.1042/BST20160364

**Published:** 2017-07-14

**Authors:** Robert Harvey, Veronica Dezi, Mariavittoria Pizzinga, Anne E. Willis

**Affiliations:** Medical Research Council Toxicology Unit, Lancaster Rd, Leicester LE1 9HN, U.K.

**Keywords:** epitranscriptome, RNA-binding proteins, stress granules, stress response, translation, translational reprogramming

## Abstract

The ability of mammalian cells to modulate global protein synthesis in response to cellular stress is essential for cell survival. While control of protein synthesis is mediated by the regulation of eukaryotic initiation and elongation factors, RNA-binding proteins (RBPs) provide a crucial additional layer to post-transcriptional regulation. RBPs bind specific RNA through conserved RNA-binding domains and ensure that the information contained within the genome and transcribed in the form of RNA is exported to the cytoplasm, chemically modified, and translated prior to folding into a functional protein. Thus, this group of proteins, through mediating translational reprogramming, spatial reorganisation, and chemical modification of RNA molecules, have a major influence on the robust cellular response to external stress and toxic injury.

## Introduction

Following exposure to a wide range of toxic agents, several gene regulation programmes are initiated to orchestrate the appropriate cellular response. An essential part of the cell stress response occurs post-transcriptionally, with over 90% of all mRNAs being regulated at this level. Post-transcriptional control is achieved through combinatorial sets of RNA-binding proteins (RBPs) which, in conjunction with post-translational modification programmes, recognise RNA regulatory motifs or regions of secondary structure within RNAs [1]. RBPs control gene expression through a wide range of processes. For example, proteomic and genetic screening has suggested a large co-transcriptional engagement of splicing factors in the response to toxic injury such as DNA damage [2]. In addition, following exposure to heat and cold shock, oxidative stress, hypoxia, ultraviolet light (UV) exposure, and ionising irradiation, RBPs have been shown to regulate a wide range of cytoplasmic processes such as RNA stability, translation efficiency, and localisation to cytoplasmic granules (Table 1). Moreover, the recent improvement of next-generation sequencing platforms has enabled investigation of the extensive chemical modifications of RNAs [3] and suggests that RBP-mediated modification of the epitranscriptome is a key component of the response to toxic injury [4].

**Figure 1.**
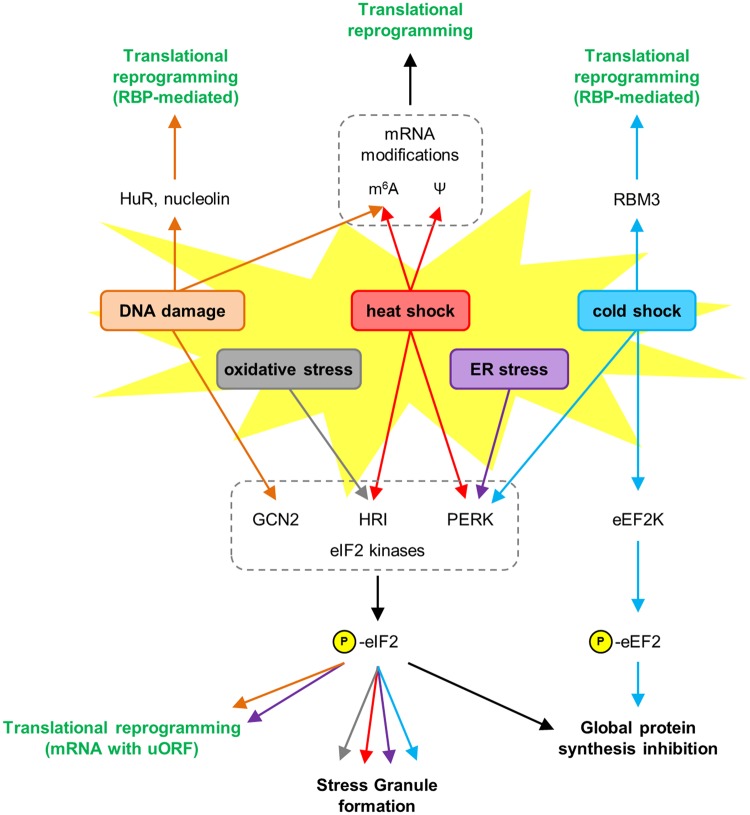
Translational reprogramming in response to cellular stress. Toxic insults, including DNA damage (orange), oxidative stress (grey), heat shock (red), ER stress (purple), and cold shock (blue), activate eIF2 kinases and phosphorylate eIF2 (p-eIF2) to induce SG formation and inhibit global protein synthesis. Translation inhibition can also be achieved via phosphorylation of eEF2 following cold shock. Translational reprogramming enables the expression of specific stress response mRNAs and can be mediated by the presence of upstream open reading frames (uORFs), chemical modifications within mRNAs, and modulation of RBP activity.

**Table 1:** RBPs modulated in response to stress List of nuclear and cytoplasmic RBPs identified to be modulated in response to cold and heat shock, hypoxia, and DNA damage.

Stress	RBPs	References
Cold shock <35°C	CIRBP, RBM3, PABP, EIF3B, G3BP	[51,66]
Heat shock >42°C	YTHDF2, YTHDF1, TIA1, TIAR, HSP70, HSP110, G3BP, ATXN2, ATXN2L, FMRP, TDRD3	[46,60,67–69]
Hypoxia Mild: 8% O_2_ Severe: 1% O_2_	HuR, PTB, TTP, NCL, CPEBs, TIA1, TIAR, G3BP, ERBP, hnRNPs (A18, L), RBM3, CIRBP, GAPDH	[17,70,71]
DNA damage Chemotherapeutics UV IR Replication errors Oxidative metabolism	HuR, EWS, YB1, PCBP4, NCL, hnRNPs (A0, A1, A18, F, H, K, R, U-like), KSRP, PSF, RBMX, SRSF1, MDM2, DDX17, TLS/FUS, SKIP, RPL26, TAF15, AATF, BRCA1, BCLAF1, DDX39B, CDCL5, DDX5, NONO, RBM14, RBM38, RPS3, SUMO1, USP10, XRCC6, XRCC5, XRCC6, RBM3, G3BP, TIAR, FMRP, PABP1, Staufen, TDRD3	[66,68,72–78]

In this review, we will retrace fundamental notions of post-transcriptional regulation following stress and toxic injury and will highlight recent findings on the crucial contribution of RBPs in the process.

## RBP-mediated reprogramming of translation following toxic injury

Cap-dependent translation is a three-stage process, comprising initiation, elongation and termination, regulated by eukaryotic initiation and elongation factors (eIFs and eEFs). The process of initiation requires the trimeric protein complex eIF4F, composed of the scaffold protein eIF4G, the cap-binding protein eIF4E, and the DEAD-box helicase eIF4A. The interaction between eIF4G and eIF3 is required for recruitment of the ribosome, whereas the interaction between eIF4G and poly(A)binding protein (PABP) circularises the mRNA and stabilises the complex [5]. mRNA decoding occurs during the elongation stage, the rate of which is determined by tRNA- and eEF1A-dependent codon decoding, and eEF2-dependent ribosome translocation.

Although it has been proposed that translational control is mostly exerted at the initiation phase [5], recent data suggest that elongation also makes a major contribution to the overall regulation of this process [6]. Global control of protein synthesis is mediated by the phosphorylation of eIF2α, which inhibits the formation of a translationally competent ternary complex [5], dephosphorylation of eIF4E-BP, which prevents the assembly of the eIF4F complex by sequestration of the eIF4E factor [5], and phosphorylation of eEF2, which inhibits ribosome translocation [7].

Protein synthesis is one of the most energy-demanding processes within the cell. Therefore, in response to a toxic insult, cells reduce global levels of protein synthesis to conserve energy and embark on the process of translational reprogramming, which is vital for the cellular response to stress [8–10]. For example, DNA damage and ER stress have been described to inhibit global protein synthesis through the regulation of eIF2, while simultaneously enhancing the translation of mRNAs required for DNA repair and restoration of ER homeostasis, respectively [8,11]. Similarly, during hypoxia, global translation is inhibited through inactivation of eIF4E, but residual hypoxic translation is maintained by switching to the eIF4E isoform eIF4E2, which works in a complex with hypoxia-inducible factor 2α (HIF-2α) and the RBP RBM4 [12]. Since eIF4E2 is not able to bind to eIF4G1 [13], it is possible that specific 4G isoforms might also be involved in the stress response. This would not be unprecedented, given that the eIF4G homologue DAP5 has been reported to allow for translation of specific mRNAs following ER stress [14].

Currently, over 800 human RBPs have been identified [15,16], many of which regulate the translation of target mRNA in a combinatorial manner. The modulation of RBPs present on an mRNA represents an important aspect of translational reprogramming, as it leads to changes in stability and translational efficiency of the message. For example, in response to the hypoxia mimic cobalt chloride, the translation of HIF-1α mRNA was shown to be up-regulated through the combined binding of HuR and PTB [17]. Conversely, the translation of cytochrome *c* mRNA is co-ordinated by the opposing actions of HuR (translational activator) and TIA-1 (translational repressor), to maintain precise levels of the pro-apoptotic protein within the cell [18].

Intriguingly, proteomic analysis has identified RBPs as potential substrates for DNA damage response (DDR) kinases ATM and ATR [19], indicating that modulation of RBP function is of paramount importance for the reprogramming of translation after DNA damage. One of the most extensively studied RBPs, HuR, predominantly stabilises its target mRNAs and regulates cellular processes such as mRNA processing and cell-cycle regulation [20]. In response to DNA damage induced by ionising radiation, checkpoint kinase 2 phosphorylates HuR and triggers its dissociation from target mRNA. HuR target mRNAs include MDM2 (a negative regulator of p53) and Bax (pro-apoptotic protein), and dissociation of HuR reduces their stability and translational efficiency, resulting in enhanced cell survival [21]. Moreover, HuR plays a key role in enhancing the translation of specific mRNA whose protein products are required for the DDR. In response to UV radiation, HuR was shown to enhance the translation of p53 [22]. Additionally, HuR was identified to bind p21 mRNA [23], a message that is transcriptionally up-regulated by p53, presumably enhancing its stability during p53-dependent cell-cycle arrest. The RNA-binding activity of nucleolin was also shown to be up-regulated following induction of ionizing radiation (IR)- and UV-dependent DNA damage, enhancing the translation of target transcripts involved in the stress response [24]. Interestingly, DNA damage also alters the transcription of mRNAs encoding RBPs, for example the anti-apoptotic protein Staufen2 is down-regulated, resulting in the activation of cell death pathways [25].

Whereas DNA damage inhibits global protein synthesis through the inhibition of initiation, the cold shock response, such as the drop in temperature observed in hibernating animals, regulates global protein synthesis through the inhibition of elongation [26]. Importantly, two cold shock proteins, RBM3 and CIRBP, function as RBPs and are transcriptionally up-regulated during mild hypothermia [27,28]. In particular, RBM3 contributes to cold shock-induced translational reprogramming by up-regulating the translation of RTN3 mRNA during global protein synthesis inhibition [29]. Therapeutic hypothermia is a promising treatment for neurodegenerative disorders and cooling has been shown to be neuroprotective in mice [30]. Importantly, the enhanced translation of RTN3 during cooling was shown to mediate the neuroprotective properties of RBM3 [29], highlighting the clinical relevance of translational reprogramming.

In summary, RBPs play a crucial role in the regulation of translational reprogramming during cellular stress, providing an additional mechanism to mediate the translation of mRNA required for the cellular response to toxic injury.

## RBP-mediated stress-induced spatial reorganisation of RNAs

As part of the stress-induced inhibition of translation, mRNAs encoding non-essential housekeeping proteins are redirected from polysomes to stress granules (SGs) or processing bodies (P-bodies). These translationally inactive, membraneless cytoplasmic foci allow the cell to reorganise the translational pool in order to focus resources into the translation of stress response mRNAs [31,32]. In mammals, RNP granules exhibit properties of viscous liquid-like structures, or droplets, that form through phase transitions and can be tuned by several physical factors [33,34]. Although such phase transition seems to be driven by promiscuous interactions among RBPs through their low-complexity domains [34], RNA also plays a major role by serving as a platform for multivalent interactions [35–37].

P-bodies are highly conserved and generally thought to represent sites of mRNA degradation [38,39], enriched for several mRNA decay factors, including the decapping complex DCP1/DCP2, the helicase RCK/p54, and the 5′-3′ ribonuclease Xrn1 [40]. In mammals, the presence of P-bodies is not strictly dependent on conditions of stress; however, certain stimuli trigger the formation of *de novo* P-bodies, as well as changes in the size and mRNA and protein content of pre-existing ones [41–43].

In contrast to P-bodies, stress granules do not appear to function as sites of RNA degradation as much as RNA storage and generally form in response to the phosphorylation of eIF2α by stress-activated kinases [40]. A series of core components of SGs have been identified, which mainly consists of 40S ribosomal subunits and several RBPs. TIA-1 and G3BP1 are particularly important as they act as SG-nucleating proteins and their overexpression alone is sufficient to drive the assembly of ‘constitutive’ stress granules [44–46]. Other canonical components of SGs include PABP and several translation initiation factors, such as eIF3, eIF4, and eIF5 [41]. Therefore, transcripts are generally considered to be in a situation of stalled translation initiation while in SGs [47].

Despite the identification of such core elements, the protein and mRNA contents of SGs change depending on the type of stress stimulus. For example, following heat shock, the bulk of cellular mRNA is redirected to SGs, with the exception of transcripts encoding HSP70 proteins [48], whereas HSP90 mRNAs are excluded from SGs induced by treatment with the oxidative stress-inducing agent sodium arsenite (SA) [49]. Moreover, the chaperone HSP27 is present in granules induced by heat shock but not by SA or UV [46]. Different isoforms of the same protein can also be differentially localised. For example, eIF4E1 is found in both P-bodies and SGs following either SA treatment or heat shock, while eIF4E2 localises to P-bodies only following SA treatment and to both P-bodies and SGs following heat shock [49]. Moreover, following exposure to SA and UV, eIF4E3A is exclusively found in SGs, whereas eIF4E3B does not localise to either P-bodies or SGs [49].

Recently, the protein composition and assembly dynamics of human SGs induced by a plethora of stimuli have been examined, providing an unprecedentedly detailed characterisation of stress-specific differences [50]. Treatment with SA was found to result in the formation of canonical foci containing poly(A) mRNAs, as well as all the constitutive RBPs and factors described above. Poly(A) mRNAs were also found to accumulate in SGs induced by hyperosmotic stress, heat shock, and ER stress, but not in UV radiation-induced foci. UV-induced granules also contain less eIF3b and eIF4G, suggesting that they might be distinct from SGs. As expected, all the stresses examined promote accumulation of G3BP1 and TIA-1 [50].

In addition to differences in SG composition, SGs also differ in mechanisms of formation. Indeed, phosphorylation of eIF2α is necessary for the formation of SGs and the inhibition of translation following oxidative and ER stress, but is not required for hyperosmotic pressure-induced translational repression and SG formation [50]. Remarkably, specific kinases phosphorylate eIF2α following different stresses. The formation of SGs and translation inhibition rely on the activity of HRI following SA treatment, or PERK following ER stress [50] and cold shock [51]. Conversely, heat shock seems to achieve translation repression and formation of SGs by activating multiple eIF2α kinases [50]. UV-induced foci rely both on eIF2α phosphorylation-dependent, via GCN2, and -independent mechanisms. In addition to phosphorylation of eIF2α, other mechanisms have been implicated in the formation of SGs. Among these are the down-regulation of mTOR and the disruption of the eIF4F complex, either by impairing the function of eIF4A or by interfering with the interaction between eIF4E and eIF4G [52].

It is clear, therefore, that stress-dependent RNP granules in mammals are remarkably dynamic entities and that a complex network of regulation mechanisms, both global and finely tuned, combine to enable the strategical restructuring of the translatome.

## RBP-mediated modification of the epitranscriptome

The relatively recent discovery of mRNA modification has opened a new realm of post-transcriptional gene regulation in eukaryotes [4]. mRNA modifications include 7-methylguanosine (m^7^G), *N*^1^-methyladenosine (m^1^A), 5-methylcytosine (m^5^C), *N*^6^-methyladenosine (m^6^A), and pseudouridine (Ψ), which combine to form the epitranscriptome. These modifications provide an additional layer of information to regulate protein synthesis by altering the translation efficiencies of individual messages [53].

Capping of newly transcribed pre-mRNAs represents a crucial event as transcripts are predominantly translated in a cap-dependent manner. Most capped mRNAs are modified by the addition of a single methyl group at N7 (m^7^GpppN or simply m^7^G) in a reaction catalysed by RNA guanine-7 methyltransferase (RNMT). The activity of RNMT is enhanced following mitosis by CDK1-cyclin B1: mRNA capping and translation initiation are therefore co-ordinated with G1 phase transcription [54]. Accordingly, RNMT can be indirectly regulated by stresses which induce cell-cycle arrest by modulating either CDK1-cyclin B1 activity [55] or its cellular localisation [56].

The process of reversible mRNA methylation, such as m^6^A, is carried out by RBPs termed writers, erasers, and readers [57]. At present, four components of the mammalian m^6^A writer complex (methyltransferase-like 3, METTL3; METTL14; Wilms tumour 1-associated protein, WTAP; and KIAA142) and two eraser proteins (fat mass and obesity-associated protein, FTO and AlkB homologue 5, ALKBH5) have been identified. The number of known reader proteins is wider and includes members of the YT521-B homology (YTH) domain-containing protein and the heterogeneous nuclear ribonucleoprotein (hnRNP) protein families [4]. m^6^A methylation promotes mRNA translation through distinct mechanisms. For example, YTHDF1 increases the efficiency of cap-dependent translation of m^6^A-modified mRNAs by recruiting several eIFs to their 3′-UTR (untranslated region), whereas METTL3 enhances cap-independent, eIF3-dependent translation of a subset of mRNAs by promoting the addition of m^6^A in the 5′-UTR [58].

It has been shown that m^6^A modification of mRNAs is important in response to cell stress and toxic injury and can dictate cell fate. For example, following SA-initiated oxidative stress, the reader protein YTHDF1 localises to stress granules bound to stalled translation complexes, but promotes post-stress recovery by reinstating protein synthesis. During heat shock, YTHDF2 localises to the nucleus, inhibits FTO binding, and promotes 5′-UTR methylation. As a consequence, subsets of mRNAs produced during heat shock carry more m^6^A marks within their 5′-end, a modification which allows an increase in their translation through cap-independent mechanisms, overcoming global translation suppression [59,60]. Hypoxic stress induces HIF-dependent ALKBH5 expression and subsequent removal of m^6^A from the transcripts encoding the pluripotency factor NANOG, leading to increased NANOG expression [57].

Ψ forms a more stable base pair with adenosine than its isomer uridine [61] and, as a consequence, affects RNA stability and mRNA translation. Pseudouridylation of spliceosomal small nuclear RNAs and ribosomal RNAs has been well described. However, previous reports show that mRNA is also pseudouridylated under a range of conditions, including cell stress [62,63]. Pseudouridylation could enhance the synthesis of specific stress response proteins by allowing alternative decoding and increasing translational efficiency of a subset of transcripts [64]. Alternatively, as pseudouridylation stabilises RNA structures, it has been suggested that this may influence the translatome following heat shock [63]. Interestingly, it has been shown recently that incorporating N1-methyl-pseudouridine (N1mΨ) nucleotides into mRNAs increases ribosome pausing and density, suggesting that, on these modified mRNAs, initiation is increased through either enhanced ribosome recycling on the same mRNA or enhanced ribosome recruitment [65].

## Concluding remarks

For many years, mainly due to experimental limitations, the study of cellular responses to external stresses has focused on the transcriptional level. However, recent technical advances have led to an explosion in the number of identified RBPs that actively regulate the cellular response at the post-transcriptional level. Either canonical or putative RBPs assist in the global inhibition of protein synthesis by sequestering mRNA within SGs, while simultaneously enhancing the translation of specific mRNAs that are required to regulate cell fate (Figure 1). The number of identified RBPs will continue to grow as further advancements in quantitative mass spectrometry and methods of RBP isolation are made. However, one of the most important questions will concern the modulation of RBPs, and subsequent changes in RNA-binding efficiency upon exposure to external cues. Future studies will continue to enhance our current understanding of the regulation of RNA, but also have the potential to identify novel RBPs as therapeutic targets that could mark a new era of therapeutic treatments.

## Abbreviations

ALKBH5: AlkB homologue 5
eEF: eukaryotic elongation factor
eIF: eukaryotic initiation factor
ER: endoplasmic reticulum
FTO: fat mass and obesity-associated protein
HIF: hypoxia-inducible factor
hnRNP: heterogeneous nuclear ribonucleoprotein
HSP: heat shock protein
HuR: Hu antigen R
IR: ionizing radiation
m^6^A: N^6^-methyladenosine
METTL: methyltransferase-like
mTOR: mechanistic target of rapamycin
PABP: poly(A)-binding protein
PTB: polypyrimidine tract-binding protein
P-bodies: processing bodies
RBP: RNA-binding protein
RNMT: RNA guanine-7 methyltransferase
SA: sodium arsenite
SGs: stress granules
UTR: untranslated region
UV: ultraviolet light
YTH: YT521-B homology
Ψ: pseudouridine.
